# Supplementary material for: High-Recovery Desalting Tip Columns for a Wide Variety of Peptides in Mass Spectrometry-Based Proteomics
Source: Anal Chem. 2024 Dec 16;96(52):20390–7. doi: 10.1021/acs.analchem.4c03753 (PMC11696827; doi:10.1021/acs.analchem.4c03753)
Supplement: Supplementary file 1 — ac4c03753_si_001.pdf [file ac4c03753_si_001.pdf]

Supplemental Information

High-Recovery Desalting Tip Columns for a Wide Variety of Peptides in Mass Spectrometry-based Proteomics

Eisuke Kanao,[1,2,*] Shunsuke Tanaka,[1,*] Ayana Tomioka,[1] Kosuke Ogata,[1] Tetsuya Tanigawa,[1] Takuya Kubo,[3] Yasushi Ishihama[1,2]

1) Graduate School of Pharmaceutical Sciences, Kyoto University, Kyoto 606–8501, Japan
2) Laboratory of Proteomics for Drug Discovery, National Institute of Biomedical Innovation, Health and Nutrition, Ibaraki, Osaka 567-0085, Japan.
3) Graduate School of Engineering, Kyoto University, Katsura, Nishikyo-ku, Kyoto 615-8510, Japan

*These two authors contributed equally to this work.

Correspondence and requests for materials should be addressed to E.K. and Y.I.
<u>email</u>
E.K. (kanao.eisuke.7s@kyoto-u.ac.jp)
Y. I. (yishiham@pharm.kyoto-u.ac.jp)

Keywords: Peptide purification, Bottom-up proteomics, StageTip, Sponge-like polymer

<u>Table of Contents</u>

Figure S1. SEM images of the cross-section surface of SPM-tip and SDB-XC-tip.
Figure S2. Comparison of the number of identified peptides binned by retention time between ChocoTip and SDB-XC-tip.
Figure S3. Distribution of amino acid residues of unique peptides and commonly identified peptides between ChocoTip and SDB-XC-tip.
Figure S4. LC/TIMS/Q/TOF analysis of desalted peptides with ChocoTip and SDB-XC-tip.
Figure S5. Recovery comparison between ChocoTip and SDB-XC-tip for purified peptides.
Figure S6. Effect of the number of pieces of Empore SDB-XC disk in StageTip on peptide recovery.
Figure S7. Effect of ACN concentration in the elution buffer on peptide recovery with SDB-XC-tip.
Figure S8. Desalting performance of Stacking-SPM-tip.

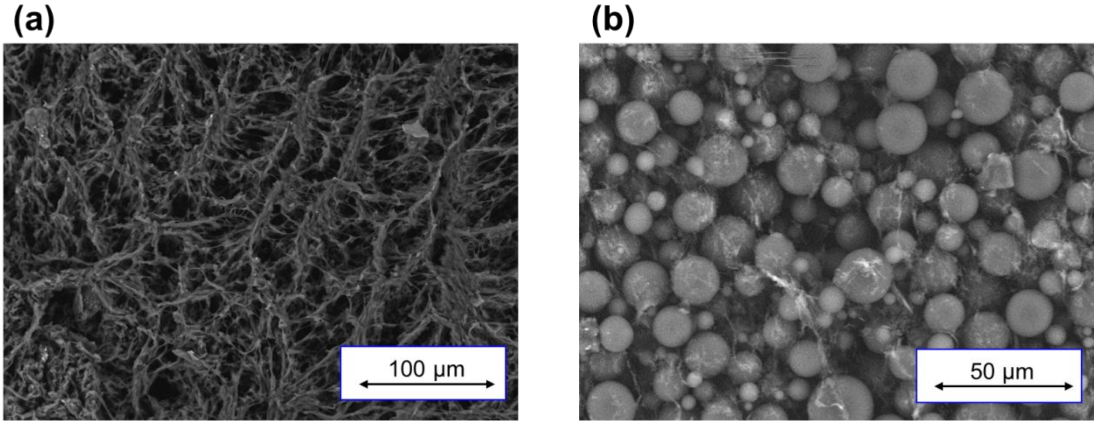

Figure S1. SEM images of the cross-section surface. (a) SPM-tip, (b) SDB-XC-tip.

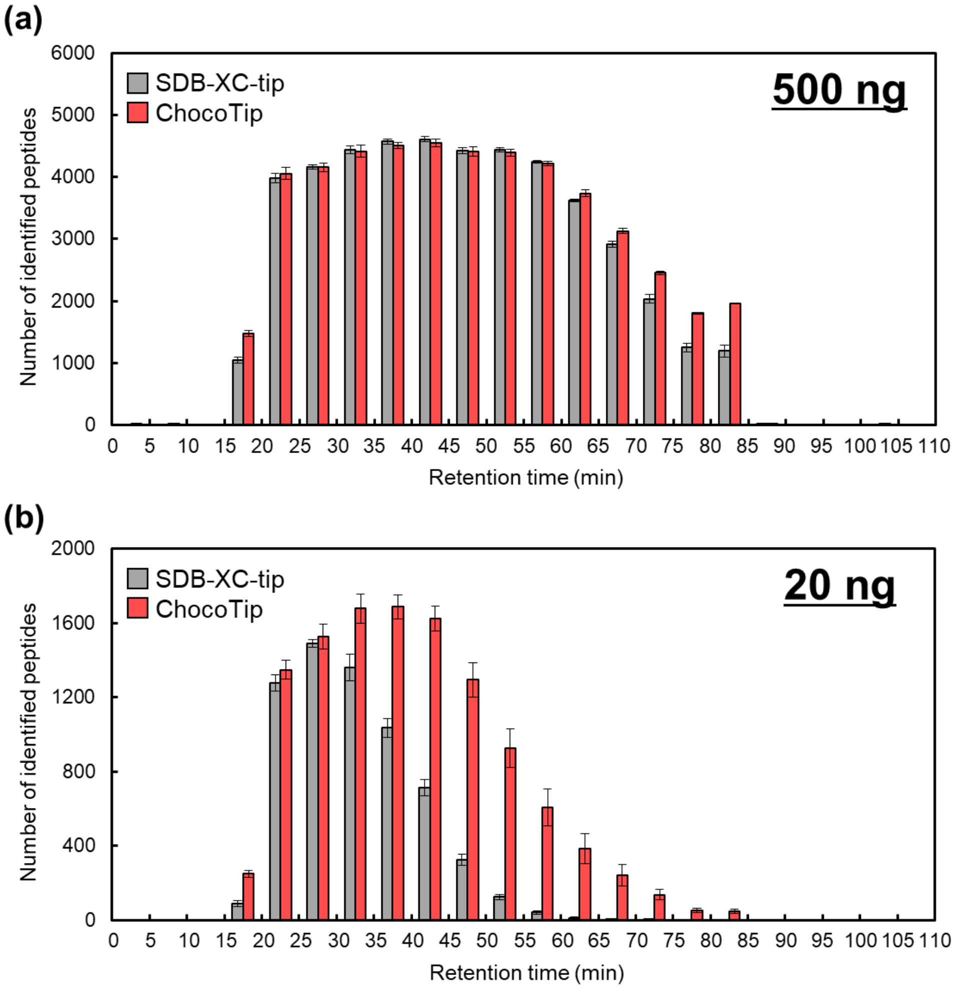

Figure S2. Comparison of the number of identified peptides binned by retention time between ChocoTip and SDB-XC-tip. StageTips were used for desalting (a) 500 ng or (b) 20 ng of tryptic peptides from HeLa cell lysates, and a sample equivalent to (a) 250 ng or (b) 10 ng of peptides was injected into the LC/MS/MS system. The error bars indicate the SDs of triplicate analyses with three StageTips.

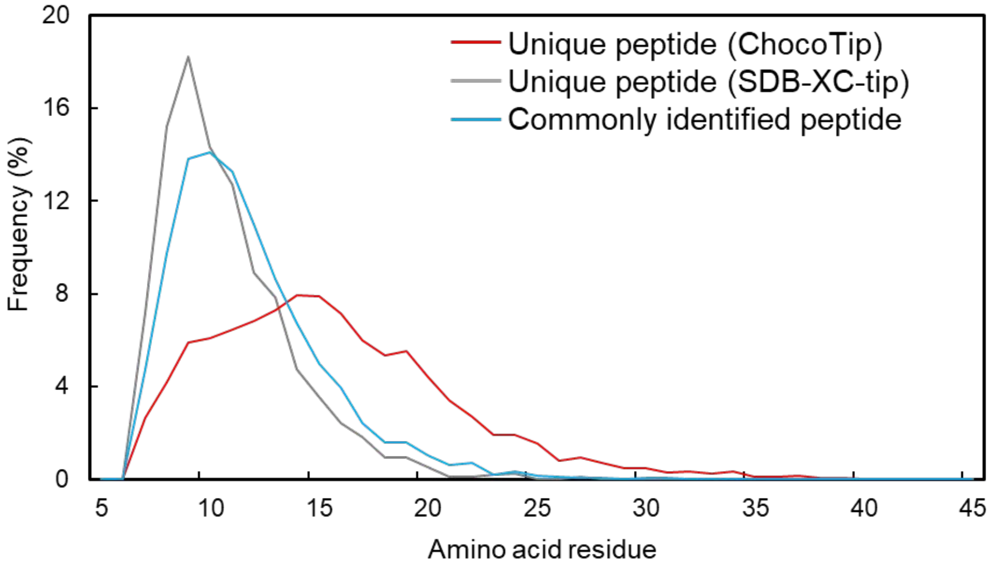

Figure S3. Distribution of amino acid residues of unique peptides and commonly identified peptides between ChocoTip and SDB-XC-tip. This data was analyzed using the same datasets as employed for Figure 2b.

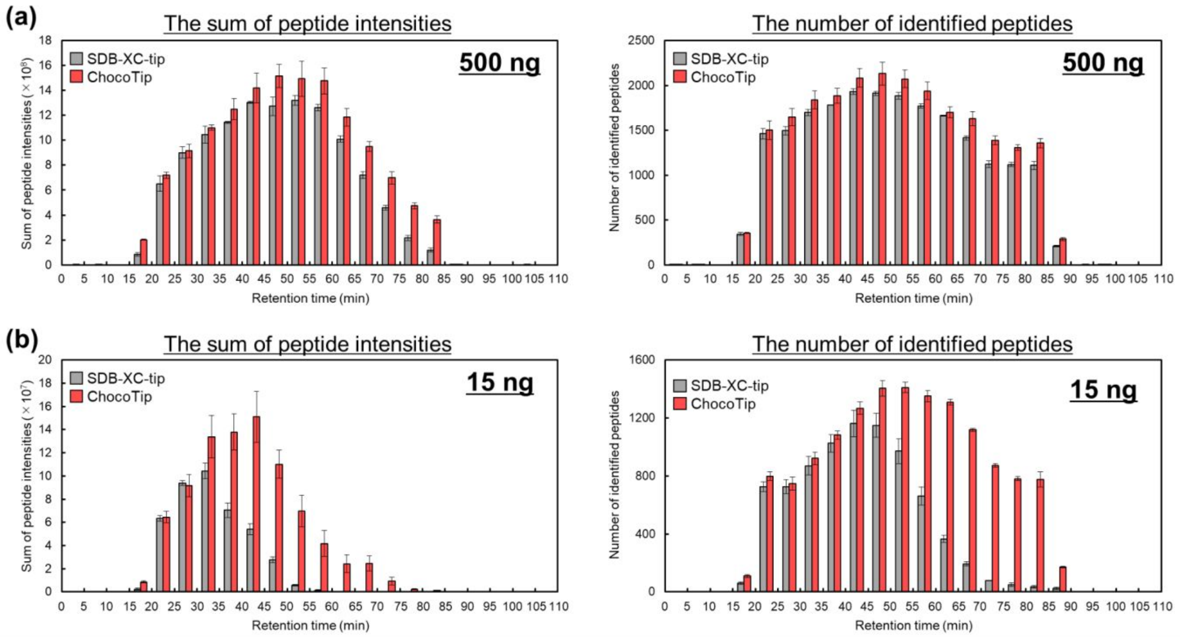

Figure S4. LC/TIMS/Q/TOF analysis of the desalted peptides with ChocoTip and SDB-XC-tip. The sum of peptide intensities and the number of identified peptides were binned by retention time. StageTips were used for desalting (a) 500 ng or (b) 15 ng of tryptic peptides from HeLa cell lysates, and a sample equivalent to (a) 250 ng or (b) 10 ng of peptides was injected onto the LC/MS/MS system. The error bars indicate the SDs of triplicate analyses with three StageTips.

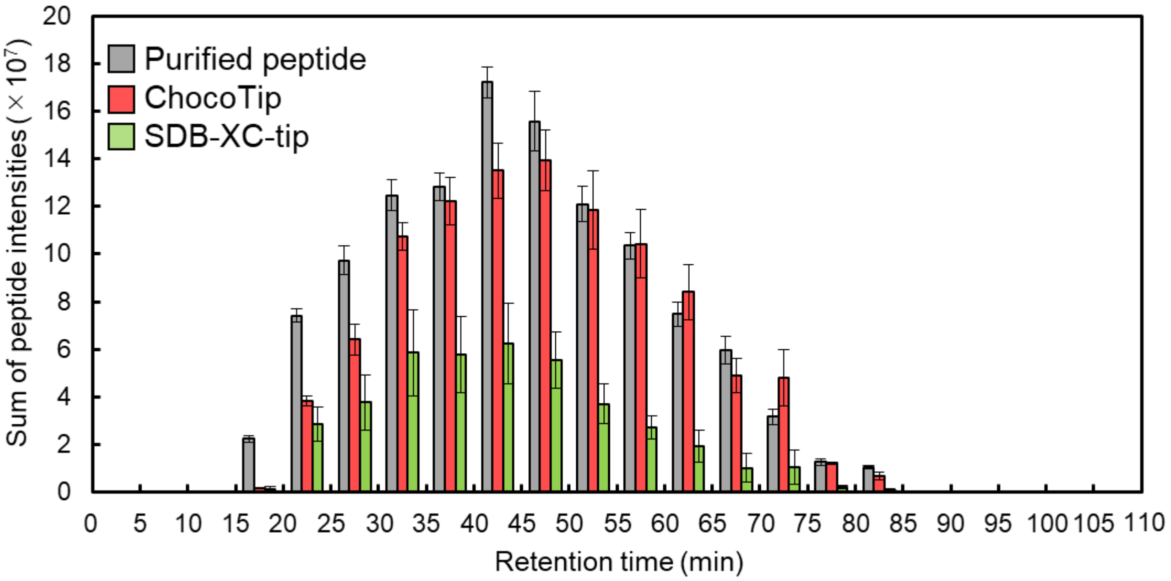

Figure S5. Recovery comparison between ChocoTip and SDB-XC-tip for purified peptides. Peptides from HeLa cell lysates were first purified with ChocoTip. The purified peptides were then subjected to a second desalting using StageTips with 20 ng of peptides, and samples equivalent to 10 ng before and after 2nd desalting were injected onto the LC/TIMS/Q/TOF system. The error bars represent the SDs from triplicate analyses using three different StageTips. The median recovery of each of the 3230 purified peptides using ChocoTip was 94.9%, while the median recovery of each of the 1273 peptides using SDB-XC-tip was only 52.2%.

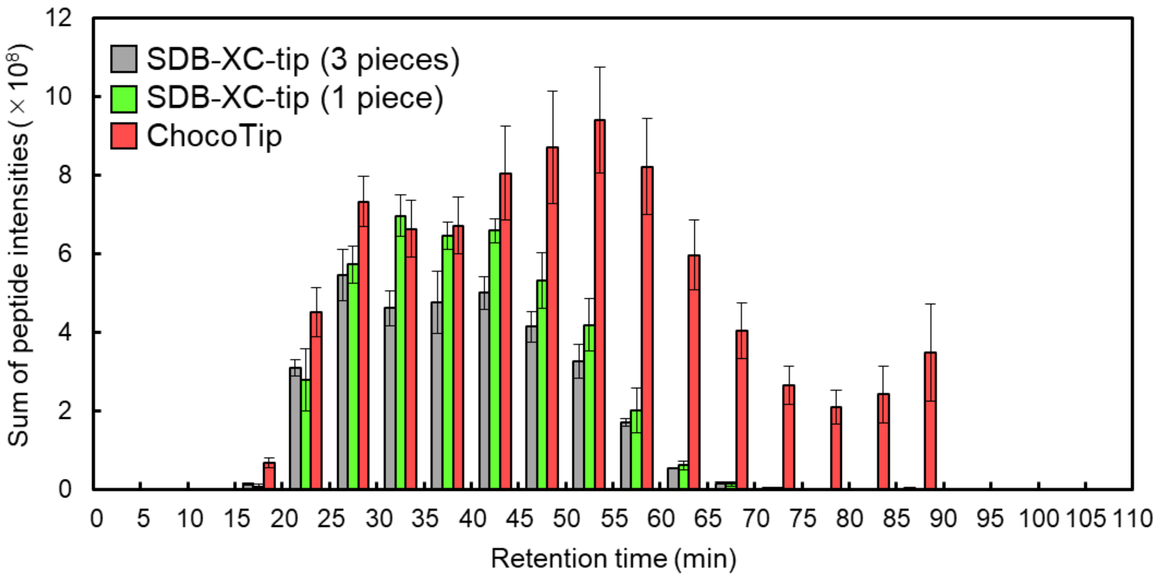

Figure S6. Effect of the number of pieces of Empore SDB-XC disk in StageTip on peptide recovery. The sum of peptide intensity was binned by retention time. StageTips were used for desalting 20 ng of tryptic peptides from HeLa cell lysates, and a sample equivalent to 10 ng of peptides was injected into the LC/MS/MS system. The error bars indicate the SDs of triplicate analyses with three StageTips.

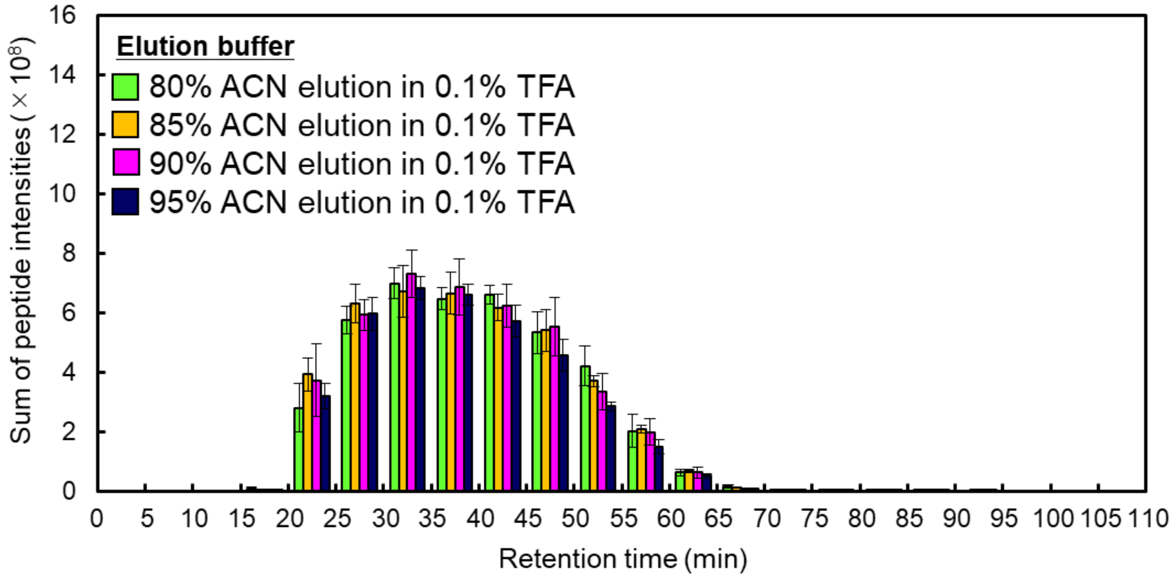

Figure S7. Effect of ACN concentration in the elution buffer on peptide recovery with SDB-XC-tip. SDB-XC-tips were manufactured by stamping out a single piece of the Empore SDB-XC disk with a 16-gauge syringe needle and packing it into a 200 µL pipette tip. The sum of peptide intensity and the number of identified peptides were binned by retention time. SDB-XC-tips were used for desalting 20 ng of tryptic peptides from HeLa cell lysates, and a sample equivalent to 10 ng of peptides was injected into the LC/MS/MS system. The error bars indicate the SDs of triplicate analyses with three SDB-XC-tips.

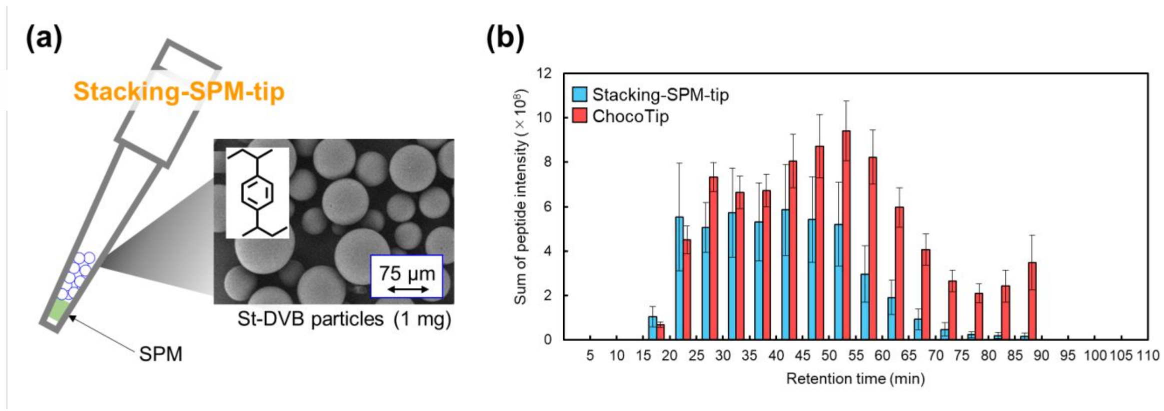

Figure S8. Desalting performance of Stacking-SPM-tip. (a) Schematic diagram of stacking-SPM-tip. Stacking-SPM-tip was prepared by wet-packing 1.0 mg of St-DVB particles into SPM-tip. (b) Comparison of the number of identified peptides binned by retention time between ChocoTip and Stacking-SPM-tip. StageTips were used for desalting 20 ng of tryptic peptides from HeLa cell lysates, and a sample equivalent to 10 ng of peptides was injected into the LC/MS/MS system. The error bars indicate the SDs of triplicate analyses with three StageTips.
